# A decision-theoretic framework for uncertainty quantification in epidemiological modelling

**DOI:** 10.1093/aje/kwag154

**Published:** 2026-07-08

**Authors:** Nicholas Steyn, Cathal Mills, Vik Shirvaikar, Freddie Bickford Smith, Christl A Donnelly, Kris V Parag

**Affiliations:** Department of Statistics, University of Oxford, Oxford, OX1 3LB, United Kingdom; Department of Statistics, University of Oxford, Oxford, OX1 3LB, United Kingdom; Pandemic Sciences Institute, University of Oxford, Oxford, OX3 7DQ, United Kingdom; Department of Statistics, University of Oxford, Oxford, OX1 3LB, United Kingdom; Department of Statistics, University of Oxford, Oxford, OX1 3LB, United Kingdom; Department of Statistics, University of Oxford, Oxford, OX1 3LB, United Kingdom; Pandemic Sciences Institute, University of Oxford, Oxford, OX3 7DQ, United Kingdom; Department of Engineering, King’s College London, London, WC2R 2LS, United Kingdom; MRC Centre for Global Infectious Disease Analysis, Imperial College London, London, W12 0BZ, United Kingdom

**Keywords:** Bayesian statistics, data science, epidemiologic methods, infectious disease epidemiology, machine learning (ML), missing data, statistical inference, uncertainty quantification

## Abstract

Estimating, understanding, and communicating uncertainty is fundamental to statistical epidemiology, where model-based estimates regularly inform real-world decisions. However, sources of uncertainty are rarely formalised, and existing classifications are often inconsistent. This lack of structure hampers interpretation, model comparison, and targeted data collection. Connecting ideas from machine learning, information theory, experimental design, and health economics, we present a first-principles decision-theoretic framework that defines uncertainty as the expected loss incurred by making an estimate using incomplete information, arguing that this is a practically relevant definition for epidemiology. We show how reasoning about future data leads to a notion of expected uncertainty reduction, which induces formal definitions of *reducible* and *irreducible* uncertainty. We illustrate our approach with a simple worked example and a case study of SARS-CoV-2 wastewater surveillance in Aotearoa New Zealand, estimating the uncertainty reduction if wastewater surveillance were expanded to the full population. We then connect our framework to relevant literature from adjacent fields, showing how it unifies and extends many of these ideas. Our article serves as a gateway for applying a wide range of approaches to epidemiological models. Altogether, our framework provides a foundation for more reliable, consistent, and policy-relevant uncertainty quantification in infectious disease epidemiology.

## Introduction

Statistical epidemiology seeks to learn about infectious disease dynamics by analyzing data, often by fitting models.^1^ These models are used for inference (estimating unobservable quantities such as parameters or latent variables) and/or prediction (estimating hypothetically observable data). Such estimates frequently inform real-world decision-making and public health planning.^2^ In addition to producing estimates, the modeler typically quantifies uncertainty, for example, by presenting confidence intervals, credible intervals, or other uncertainty measures.^3^

It is natural to ask where this uncertainty originates, how to quantify it, and how it might be reduced. Beyond its theoretical interest, a robust understanding of uncertainty is essential for determining which aspects of pathogen transmission to model,^4^ for guiding real-world decision-making,^5^ for planning data collection,^6^ and for communicating results.^3^ Decision-theoretic approaches have been shown to enhance the credibility of using uncertain modelling outputs in these scenarios.^7^^,^^8^

Despite its importance, there is little agreement on how to systematically conceptualise and categorise uncertainty in epidemiological models, a lack of consensus also seen in other fields.^9^ The epidemiological literature on uncertainty is fragmented, with inconsistent definitions, terms, and classifications (eg,^10^^,^^11^^,^^12^). Discussions can be qualitative, lacking the mathematical formalism necessary for reproducibility, comparison between studies, and practical application.

This paper assumes uncertainty arises from making an estimate with incomplete information.^13^^,^^14^ Any decision would be fully informed if we had access to “complete data,” where the precise definition of “complete data” depends on the model and problem setting, but uncertainty fundamentally arises from the fact that such data are never available. This leads to an intuitive decision-theoretic framework for uncertainty quantification which also induces a practical but rigorous notion of *reducible* and *irreducible* uncertainty.

Our aim is to connect principles from machine learning,^9^ information theory,^15^^,^^16^ health economics,^17^^,^^18^^,^^19^ and other fields with the practical needs of epidemiological modelling, providing a gateway that facilitates the access and uptake of these ideas. We adapt and integrate these ideas into a framework built around four principles while also reflecting on broader conceptualisations. Our framework unifies existing concepts, such as value-of-information (VOI),^17^^,^^18^^,^^19^ Bayesian experimental design,^6^^,^^20^^,^^21^^,^^22^ and scoring rules,^7^^,^^23^ in an uncertainty-first form tailored towards epidemiology.

We focus on uncertainty arising within a chosen model and do not attempt to measure the mismatch between the model and the true data-generating process. Statements made about uncertainty here apply to the real world only if the model is correctly specified. However, our framework is a necessary step towards broader uncertainty quantification and we signpost extensions in the discussion.

We first present our decision-theoretic approach as four principles, using two conceptual examples: estimation of infection prevalence (including a simple numeric worked example) and estimation of the instantaneous reproduction number $R_{t}$.^24^  *An application to wastewater surveillance* applies this framework to a real-world model of wastewater surveillance for SARS-CoV-2 in Aotearoa New Zealand. *Related frameworks* discusses how this framework connects ideas from adjacent fields. The article then concludes with a discussion.

## A decision-theoretic framework

Model-based uncertainty has two components: a reducible component that can be resolved by collecting additional data, and an irreducible component that cannot.^13^^,^^14^ For inference, this missing information can be understood as the (possibly infinite) additional data that, if observed, would allow us to precisely determine any (identifiable) target quantity. For prediction, this view separates a reducible component of uncertainty that vanishes with unlimited data (a notion of parametric uncertainty) from a corresponding irreducible component. We describe our approach by stating four principles, motivating them with two conceptual examples.

This missing-information view is central to the Bayesian perspective. In Bayesian inference, we start by describing how all unknowns behave together (by creating a joint prior distribution), and then update this description to reflect the data we observe (resulting in a posterior distribution).^25^ The remaining uncertainty is a direct consequence of data we have not observed. We can handle this missing information indirectly, by updating beliefs about model parameters,^26^ or directly, by building a model for the missing data themselves.^13^^,^^27^^,^^28^ While this perspective is foundational to Bayesian inference, our framework is not restricted to Bayesian methods.

For example, consider estimating infection prevalence *θ * (Figure 1). If infection statuses for the full population were known, there would be no uncertainty about *θ *. With only a subset tested, a binomial model treats uncertainty as arising from an infinite unobserved population, while a hypergeometric model treats it as directly arising from the finite unobserved population. In both cases, the model dictates how uncertainty arises from the missing information.

**Figure 1.**
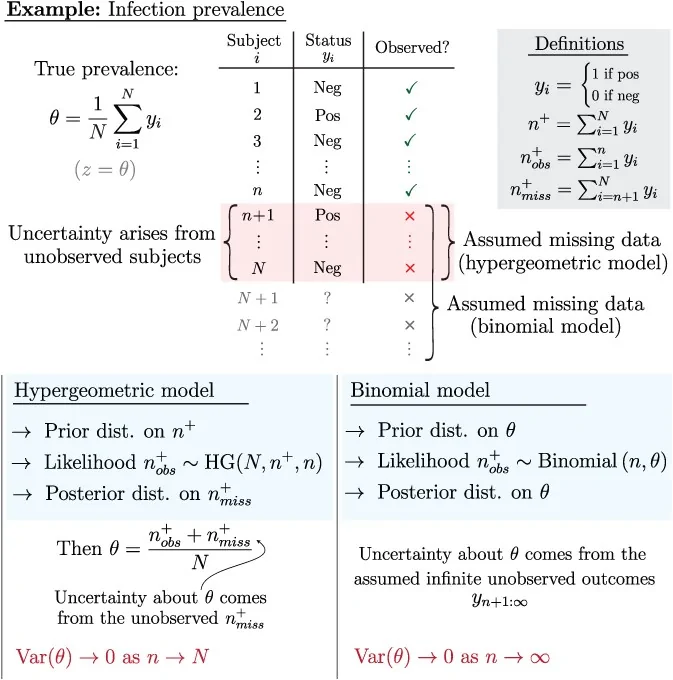
Uncertainty associated with estimating infection prevalence (here, *z = θ *). Assume we test *n* subjects out of a population of size *N* and observe $n^{+}_{obs}$ positive results. The missing data are the number of positive results $n_{miss}^{+}$ in the untested $N\!-\!n$ subjects. A popular approach is to place a prior distribution on *θ * and model the observed data using a binomial distribution, resulting in a posterior distribution whose variance goes to *0* as $n \to \infty $. In this example, the assumed missing data are the infection status of an infinite number of untested individuals. An alternative approach is to place a prior distribution on the true total number of infected subjects $n^{+}$ and model the observed data using a hypergeometric distribution, directly resulting in a posterior distribution on $n_{miss}^{+}$. By setting $\theta = \frac{n^{+}_{obs} + n^{+}_{miss}}{N}$, we obtain a posterior distribution on *θ * whose variance (one possible measure of uncertainty) goes to *0* as *n → N*. In this example, the assumed missing data are the infection statuses of the finite number of untested individuals.

### Principle 1: Model-based uncertainty arises from decision-making under incomplete information

Decision theory directly relates the missing data to a real-world decision, giving rise to concrete and problem-grounded measurements of uncertainty. For simplicity, we take the decision to be making an estimate (covering both inference and prediction), where the statistical estimator performs the role of a decision rule that maps observed data to an estimate. The framework is readily extended to other types of decisions (such as whether to impose an intervention), where the decision rule maps observed data to a real-world action.

The modeler’s role is to make an estimate *a* of an unknown quantity *z*, inducing a loss *ℓ (a, z)* to be minimised. *ℓ (a,z)* encodes the real-world consequence of making an estimate *a* when the true value is *z*, a concept familiar to machine-learning modellers and statistical forecasters.^29^^,^^30^ The unknown *z* could be a model parameter (eg, $R_{t}$) or a future observation (eg, upcoming hospitalisations).

The decision-theoretic approach begins by fitting a model $p(z|y_{obs})$ to the observed data $y_{obs}$. In the prevalence example, this is the posterior distribution $p(\theta |y_{1:n})$. To aid interpretation, we represent our problem using a graph: the square represents a decision to be made, the circle represents a random quantity, and the diamond represents the resulting loss.^31^



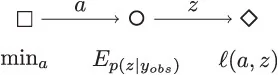



We make an estimate *a* (the square), and then observe the random *z* (the circle), inducing a loss *ℓ (a,z)* (the diamond). Identifying the *Bayes-optimal* estimate, denoted $a^{*}$, involves working backwards through this graph: starting with the loss-function, we take an expectation (that is, average) over the fitted model, and then perform a minimisation at the decision node. That is, the *Bayes-optimal* estimate $a^{*}$ minimises the expected loss $E_{p(z|y_{obs})}[\ell (a, z)]$ under the fitted model: 


(1) \begin{align*}& a^{*} = \arg\min_{a} E_{p(z|y_{obs})}[\ell(a, z)].\end{align*}

Two common choices illustrate this. If the goal is a best guess for *z* (a point estimate), a natural loss function is the squared error $\ell (a,z) = (a-z)^{2}$, which penalises estimates far from the truth. The optimal point estimate under this loss is the posterior mean. If the goal is instead to provide a full probability distribution for *z* (a probabilistic estimate), a natural loss function is the log-loss $\ell (a,z) = -\log a(z)$ (where *a* is a probability distribution), which penalises distributions under which the true value is unlikely. The optimal probabilistic estimate under this loss is the posterior distribution itself.

A key insight of DeGroot^32^ is that the minimised expected loss provides a general problem-grounded definition of uncertainty.^9^^,^^33^ Borrowing notation from Bickford Smith et al.,^9^ we measure uncertainty *h* using this minimised expected loss: 


(2) \begin{align*}& \begin{aligned} h\left[p(z|y_{obs})\right] = E_{p(z|y_{obs})}[\ell(a^{*}, z)]. \end{aligned}\end{align*}

Multiple popular measures of uncertainty arise from this setup.^9^^,^^18^^,^^33^ For a point estimate with quadratic loss, uncertainty is measured by the posterior variance: 


(3) \begin{align*}& h\left[p(z|y_{obs})\right] = \textrm{Var}_{p(z|y_{obs})}(z).\end{align*}

For a probabilistic estimate with log-loss, uncertainty is measured by the posterior (differential) entropy: 


(4) \begin{align*}& h\left[p(z|y_{obs})\right] = \textrm{H}\left[p(z|y_{obs})\right].\end{align*}

Short derivations of these are given in Appendix S1. We illustrate both cases for prevalence estimation in Figure 2, and the first case in the following worked example.

**Figure 2.**
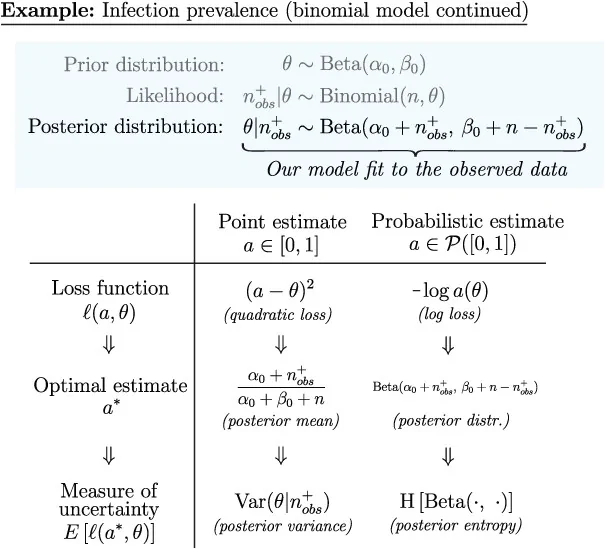
**Principle 1**. Two measures of uncertainty in the infection prevalence example with the binomial model (*z = θ * is the estimand). For simplicity, we only show the binomial model. If we use a conjugate $\textrm{Beta}(\alpha _{0}, \beta _{0})$ prior distribution, then the posterior distribution is $\textrm{Beta}(\alpha _{0} + n_{obs}^{+}, \beta _{0} + n - n_{obs}^{+})$. If we make a point estimate of *θ * under the quadratic loss function, then the Bayes-optimal estimate is the posterior mean and uncertainty can be measured using the posterior variance. If we make a probabilistic estimate of *θ * under the log-loss, then the Bayes-optimal estimate is the posterior distribution itself and uncertainty can be measured using the posterior (differential) entropy.

**Worked example (Principle 1).** We want to estimate infection prevalence *θ * in a town of $N = 10{,}000$ people. We test *n = 100* at random and find $n_{obs}^{+} = 10$ positive. Using a uniform prior distribution and a binomial model, the posterior distribution is Beta(11, 91). If we use a quadratic loss (penalising squared estimation error), our Bayes-optimal estimate is the posterior mean (*11/102 ≈ 0.108*) and the measure of uncertainty is the posterior variance (*≈ 0.00093*), the expected squared error, which is a concrete and interpretable measure of uncertainty when we are averse to outliers. That is, we estimate about 1,080 of the 10,000 residents are infected, with a typical error of $\sqrt{0.00093} \approx 0.03$ (3 percentage points, or approximately 300 people). Alternatively, under an absolute-error loss $\ell (a,\theta ) = |a - \theta |$, the Bayes-optimal estimate is the posterior median (*≈ 0.105*) and uncertainty is the expected absolute error (*≈ 0.024*), or about 2.4 percentage points (240 people). This is more appropriate if we are not especially averse to large errors.

The measure of uncertainty is determined by the loss function, so alternative functions can capture different priorities about estimation error. When estimating $R_{t}$, for example, authorities may be risk averse, wanting to penalise underestimates more than overestimates. Here, we could use an asymmetric loss function, or target tail risk with a quantile loss function,^34^ yielding problem-specific uncertainty measurements that reflect user-specific risk preferences. These problem-specific measurements can be used to guide decision-making, data-collection, and model comparison (for example).

### Principle 2: The incomplete information driving uncertainty is application-specific and determines the decomposition of reducible and irreducible uncertainty

In machine learning and other fields, data are often assumed to be drawn independently from a fixed underlying distribution: each observation is a fresh sample from the same source. Under this assumption, irreducible uncertainty (sometimes called “aleatoric” uncertainty,^35^ though definitions vary^9^) is the uncertainty that would remain even with an unlimited supply of data. In this view, the missing information corresponds to the infinitely many observations we have not yet collected.

In epidemiological transmission modelling, we only ever observe one realisation of a given epidemic, making the empirical quantification of inherent uncertainty difficult.^35^ Arguing about repeating the epidemic multiple times is of limited practical value, as this offers little insight into the uncertainty that is practically reducible. Uncertainty instead must be reduced by collecting higher-quality, or a greater quantity of, data during the epidemic.^15^

We argue that focusing on missing data $y_{miss}$ that *could practically be observed* is more useful. Specifically, we define the uncertainty reduction as the difference between the uncertainty of the model fit to the observed data ($y_{obs}$) and the uncertainty of the model fit to the full data ($y_{all} = y_{obs} \cup y_{miss}$): 


(5) \begin{align*}& \underbrace{\textrm{UR}_{z}(y_{miss})}_{\textrm{Uncertainty reduction}} = \underbrace{h\left[p(z|y_{obs})\right]}_{\textrm{Total uncertainty}} - \underbrace{h\left[p(z|y_{all})\right].}_{\textrm{Irreducible uncertainty}}\end{align*}

For example, consider estimating $R_{t}$ using the Poisson renewal model (Figure 3).^24^ Under perfect case reporting, all of the uncertainty in $R_{t}$ is irreducible as only repeated realisations of the same epidemic could improve our estimates. In the presence of underreporting, the missing data are unreported cases, and knowing these would reduce $R_{t}$ uncertainty. This illustrates a key difference between epidemiology and fields such as machine learning: we often observe only a single realisation of the epidemic (the data-generating process).

**Figure 3.**
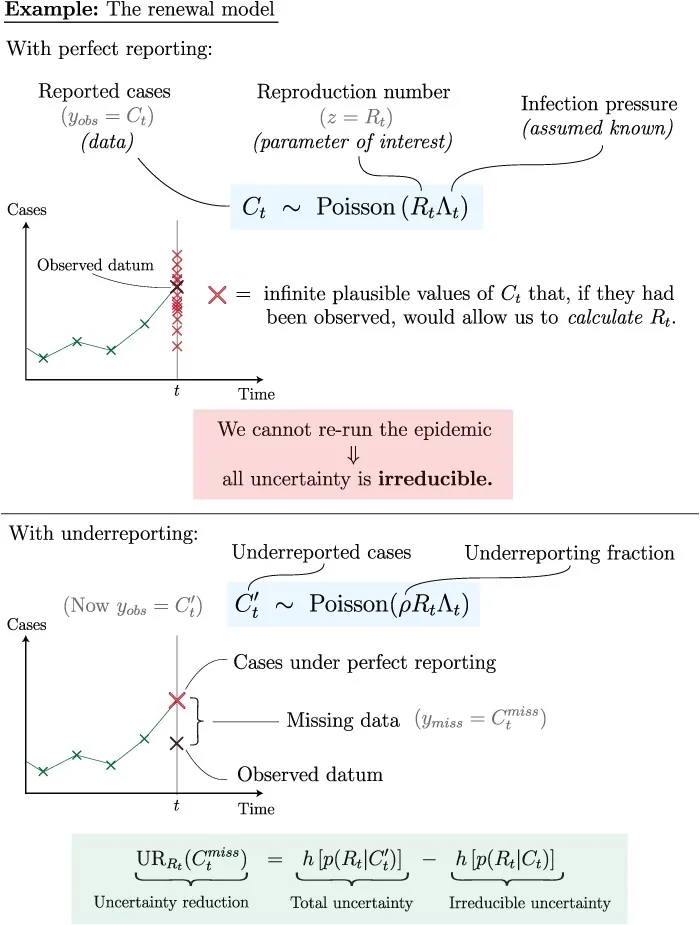
**Principle 2.** Reducible and irreducible uncertainty in the renewal model. The instantaneous reproduction number ($R_{t}$) is a key quantity in epidemiological modelling, defined as the average number of secondary infections caused by an infected individual at time *t* if they were to undergo their entire infectious period at this time.^55^ It is commonly estimated from reported case data using the Poisson renewal model pictured here.^24^^,^^56^ Even if the reported cases $C_{t}$ perfectly corresponded to the true infections (ie, no underreporting), uncertainty about $z = R_{t}$ would still exist. Under this model, uncertainty about $R_{t}$ could be eliminated only if we were to repeat the epidemic infinitely many times, thus all uncertainty is irreducible. In the presence of underreporting (*ρ *), the missing data are the unreported infections. If we knew these quantities, for example, by improving outbreak surveillance, we could reduce our uncertainty about $R_{t}$.

This construction is purposely broad. The forms of $p(z|y_{obs})$ and $p(z|y_{all})$ are unrestricted, allowing any model to be used. We also note that the form of the missing data $y_{miss}$ need not match $y_{obs}$, enabling the inclusion of different datasets in the model. We illustrate this in *An application to wastewater surveillance*, considering the inclusion of wastewater data in a model fit only to reported cases.

**Worked example (Principle 2).** Returning to our prevalence example, the missing data are the infection statuses of the $9{,}900$ untested individuals. If we could test all $10{,}000$ with a perfect test, we would know the true prevalence precisely. All of our current uncertainty (*h ≈ 0.00093*) is *reducible*. If we had an imperfect test, we will still have some uncertainty after testing everyone, which would be *irreducible*, and measured by the remaining non-zero value of *h*.

### Principle 3: To quantify reducible and irreducible uncertainty, we reason about the missing data

In some scenarios, we may know the value(s) of $y_{miss}$, for example, when including an additional already collected dataset in a model. Often, however, we do not know the value(s) of $y_{miss}$ in advance, such as when planning future data collection, or estimating the irreducible uncertainty. In these cases, we must reason about the missing data that could be observed, and consider the *expected* uncertainty reduction.

Letting $p(y_{miss}|y_{obs})$ be a predictive model describing our beliefs about the missing data, we average over all possible realisations of $y_{miss}$. Intuitively, for each possible outcome of the missing data, we calculate what our uncertainty would be if we observed that outcome, and then weight each of these by how likely we believe that outcome to be ($p(y_{miss}|y_{obs})$). Doing this to both sides of Equation 5 gives the expected uncertainty reduction: 


(6) \begin{align*} \underbrace{\textrm{E}_{p(y_{miss}|y_{obs})}\left[\textrm{UR}_{z}(y_{miss})\right]}_{\textrm{Expected uncertainty reduction}} &= \underbrace{h\left[p(z|y_{obs})\right]}_{\textrm{Total uncertainty}}\nonumber \\&- \underbrace{\textrm{E}_{p(y_{miss}|y_{obs})}\left[h\left[p(z|y_{all})\right]\right].}_{\textrm{Expected uncertainty after collecting}\ {y_{miss}}}\end{align*}

In some scenarios, the appropriate predictive distribution may be obvious. For example, in a standard Bayesian model where we plan to collect more data of the same type, our current model already tells us what those data are likely to look like, this is the posterior-predictive distribution: $p(y_{miss}|y_{obs})$. We illustrate this in the context of prevalence estimation in Figure 4. In other scenarios, this predictive distribution may be less clear. In either case, the predictive distribution is necessarily subjective.

**Figure 4.**
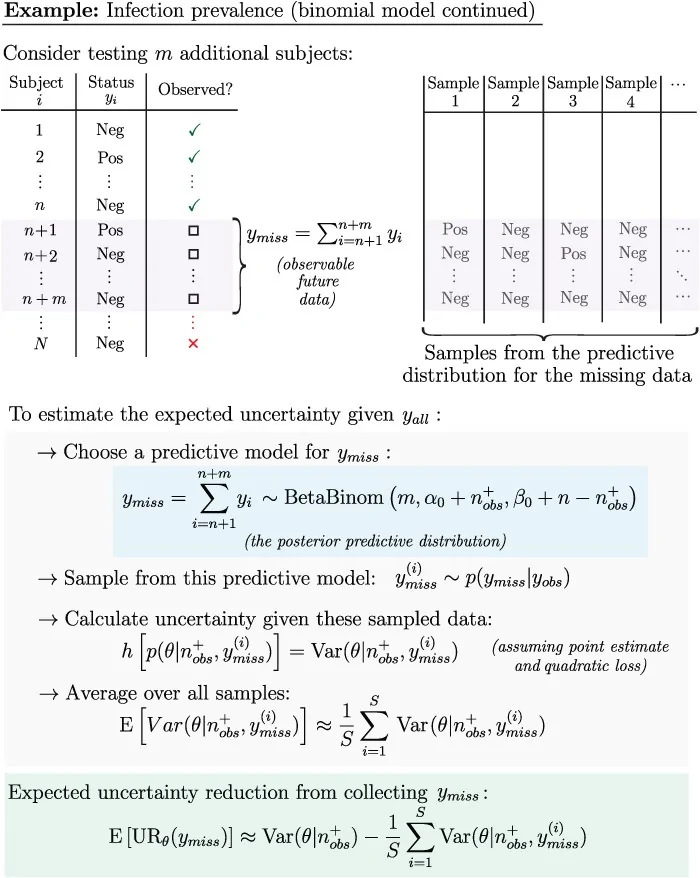
**Principle 3.** Estimating the expected uncertainty reduction in the infection prevalence example with the binomial model (*z = θ *). Consider testing *m* additional individuals for infection. The posterior-predictive distribution given a beta prior distribution and binomial likelihood is a beta-binomial distribution. We calculate the expected uncertainty reduction about *θ * after collecting $y_{miss}$ by averaging $h\left [p(\theta |y_{1:n}, y_{miss})\right ]$ (the final term in equation 6) over this predictive distribution.

**Worked example (Principle 3).** We plan to test *m = 100* additional people, but do not yet know the results. How much do we expect our uncertainty to decrease? Under our model, the number of positives among these 100 follows a beta-binomial distribution (the posterior predictive distribution). We cannot know in advance how many will test positive, but we can consider each possibility: if 8 are positive, the updated posterior is Beta(19, 183) with variance *≈ 0.00042*. If 14 are positive, it is Beta(25, 177) with variance *≈ 0.00053*, and so on. Averaging across all possible outcomes, the expected posterior variance drops from *0.00093* to approximately *0.00047*, an expected uncertainty reduction of roughly 50%.

### Principle 4: Collecting additional data is not guaranteed to reduce uncertainty in practice, but will in expectation if the predictive distribution is *coherent*

In practice, new data may contradict existing data, leading to an increase in uncertainty about *z*. While this cannot be prevented with certainty, if the predictive distribution $p(y_{miss}|y_{obs})$ is *coherent* with the fitted model, then additional data will reduce uncertainty *in expectation*.

Coherence, in this context, means that the predictive distribution and the fitted model are consistent, that is, they both make the same assumptions about the missing data. Formally, the predictive model is coherent with the fitted model if both are marginal distributions of the same joint distribution over all quantities (see Appendix S2 for the definition). Intuitively, the model used to predict the missing data should be the same model used to estimate *z*.

If both models implicitly make different assumptions, then even collecting more data can counterintuitively increase expected uncertainty. For example, when the predictive distribution is more diffuse than what the fitted model would imply, predicted observations tend to be more extreme than the fitted model expects, which can widen rather than tighten the posterior.

When coherence holds, collecting additional data is expected to reduce uncertainty (Appendix S2). If the missing data provide no new information about *z* (ie, *z* is conditionally independent of $y_{miss}$ given $y_{obs}$), then the expected uncertainty reduction is zero. Coherence is a fundamental property of Bayesian inference: standard Bayesian models automatically satisfy this condition.^25^^,^^36^

In the prevalence example, suppose we believe the prevalence among untested individuals differs from those who volunteered for testing. If we inflate the variance of the predictive distribution to account for this, but do not include this assumption in the fitted model, then the predictive model is not coherent with the fitted model, and uncertainty may increase on average.

Coherence is not a requirement of our framework and Principles 1 to 3 are valid regardless. However, without this assumption, collecting more data may not reduce uncertainty. We note that determining whether a given model satisfies coherence is model-specific and remains an area of active research.^13^^,^^37^ All four principles are summarised in Figure 5.

**Figure 5.**
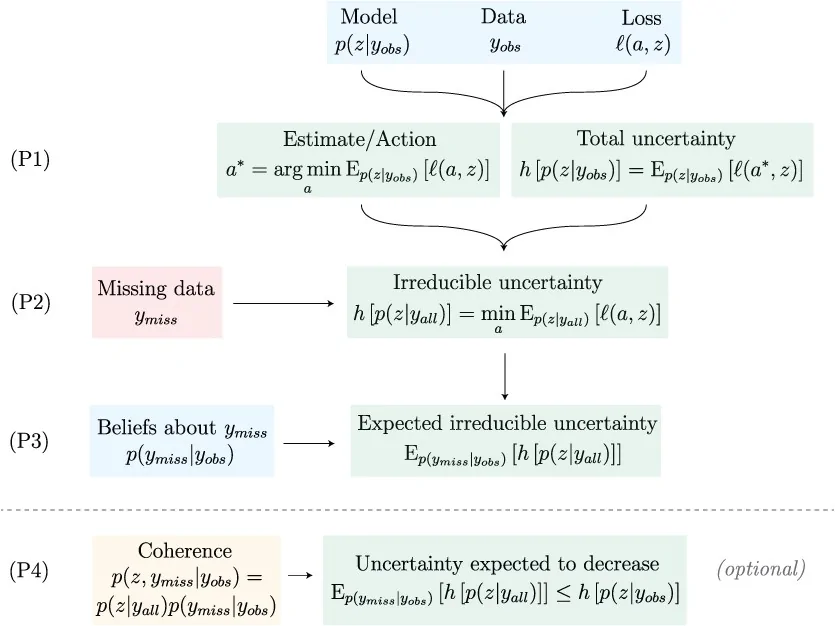
An overview of the four principles (P1 to P4) of our decision-theoretic framework. We start with a model $p(z|y_{obs})$ of the unknown quantity *z*, observed data $y_{obs}$, and a loss function *ℓ (a, z)*—the three quantities needed to make a decision under uncertainty. P1 uses the loss function to concretely relate the model and data to the real-world act of making an estimate *a* of *z*, defining the Bayes-optimal estimate $a^{*}$ and the corresponding total uncertainty *h*. P2 defines the irreducible uncertainty as the uncertainty remaining after collecting missing data $y_{miss}$. As we generally do not know the value of $y_{miss}$, P3 introduces the expected irreducible uncertainty, which is the irreducible uncertainty averaged over a predictive distribution $p(y_{miss}|y_{obs})$ of the missing data, providing a baseline of how good we can expect our estimates to possibly be. P4 provides an optional modelling condition, related to the formulation of $p(y_{miss}|y_{obs})$, under which collecting these missing data is expected to reduce uncertainty. Blue shading highlights known inputs (model, data, loss, beliefs about missing data), red shading highlights possibly unknown inputs (missing data), green shading highlights framework outputs, and yellow shading highlights the optional coherence condition.

## An application to wastewater surveillance

### How much can wastewater surveillance reduce uncertainty about $\boldsymbol{R_{t}}$?

Wastewater surveillance gained prominence during the COVID-19 pandemic as a potentially less biased source of data than traditional sources (such as reported cases) for tracking real-time pathogen transmission.^38^^,^^39^ The concentration of genomic material in wastewater does not map directly onto traditional epidemiological indicators and can be highly variable.^40^ This has led some to question the utility of wastewater data in this setting.^38^

Watson et al.^39^ constructed a joint model of wastewater and reported case data for Aotearoa New Zealand. To examine the utility of wastewater data for estimating $R_{t}$, we apply our framework to answer two questions: first, to what extent is uncertainty about $R_{t}$ reduced by including wastewater data in the model (compared to modelling reported cases only)? And second, given current wastewater sampling in Aotearoa New Zealand, how much could uncertainty about $R_{t}$ be reduced by sampling wastewater catchments covering the entire population every day? We assume decision-makers use point estimates of $R_{t}$, with a quadratic loss function deemed suitable, and therefore use variance as our measure of uncertainty. We provide full technical details of the procedure in Appendix S3.

To assess the benefit of incorporating already-observed wastewater data, we fit the model with and without these data and compute the daily uncertainty reduction (Equation 5). The “missing data” here are the daily wastewater observations, and the uncertainty reduction on each day is the difference in posterior variance of $R_{t}$ between the case-only and case-plus-wastewater model fits.

To estimate the expected uncertainty reduction from expanding surveillance to the entire population, we first fit the model to all observed data (reported cases and existing wastewater measurements), obtaining a joint posterior distribution over all model quantities. We then simulate hypothetical additional wastewater data, representing what we might observe if sampling covered the remaining population, from the posterior predictive distribution. By re-fitting the model to the combined observed and simulated data and examining the resulting posterior variance, we obtain a single draw of the uncertainty after expansion. Repeating this 100 times and averaging yields a Monte Carlo estimate of the expected uncertainty reduction (Equation 6).

We fit the model to data between 1 January 2023 and 1 March 2023 (Figure 6-A), sourced from Watson et al.^39^ Wastewater data were collected on 41 out of these 60 days (Figure 6-B), with daily catchment coverage ranging from 31,000 to 3.6 million individuals (out of 5.15 million total, Figure 6-C).

**Figure 6.**
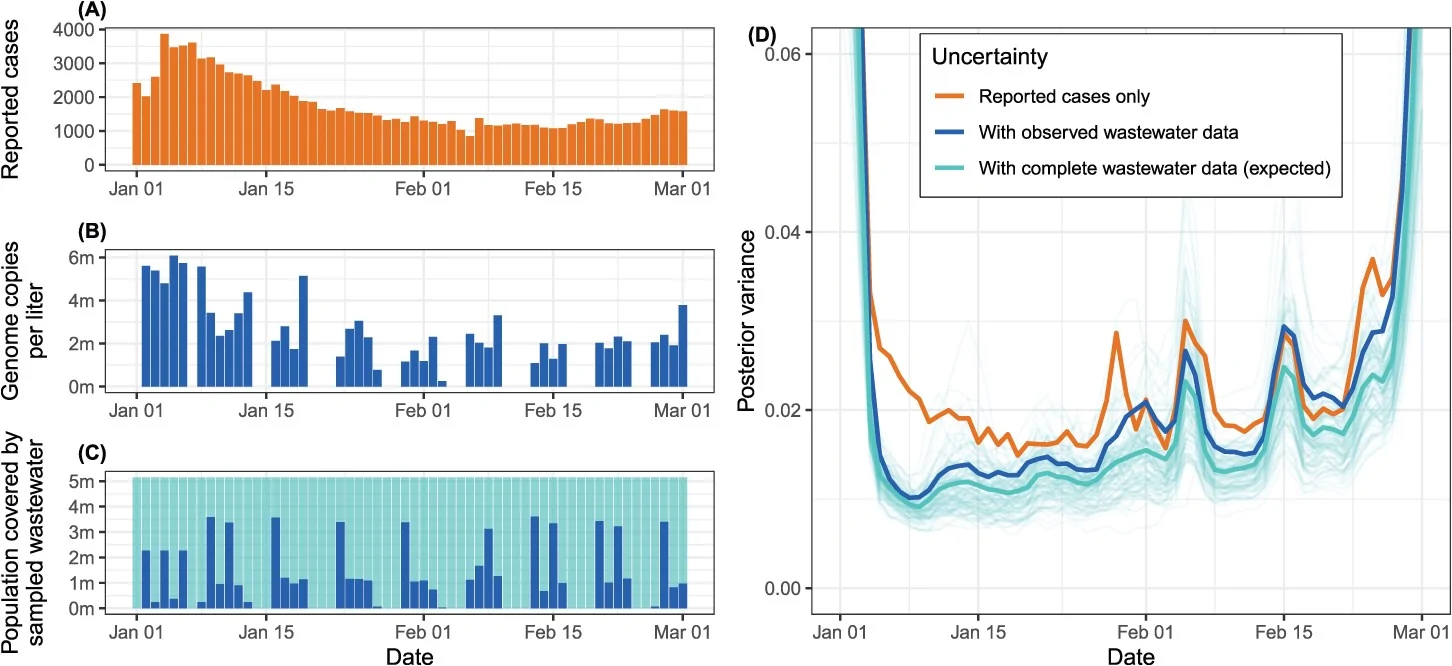
Reported case data (A), wastewater data (B), and population covered by wastewater catchment areas (C) for Aotearoa New Zealand between 1 January 2023 and 1 March 2023. The light blue region in panel (C) represents the population from which missing data could be sampled. The daily uncertainty about $R_{t}$ from the model fit to reported case data only (orange), from the model fit to both reported case data and observed wastewater data (dark blue), and the expected uncertainty after sampling wastewater data from the entire population every day (light blue) are shown in panel (D). Thin light blue lines show the posterior variance of $R_{t}$ from individual simulations of hypothetical data, demonstrating that even though the expected uncertainty reduction is positive (as we satisfy the coherence requirements), uncertainty about $R_{t}$ could still plausibly increase. Uncertainty increases at the start and end of the time period as a result of fewer data points being available to fit the model.

Including wastewater data reduces expected daily uncertainty about $R_{t}$ by an average 16.9%, with the greatest reduction being 54.4% on 7 January 2023, though some increases (up to 12.6% on 31 January 2023, 12.1% on 17 and 18 February 2023) occur on some days (Figure 6-D), highlighting that even when uncertainty is *expected* to decrease, there are instances where it may still increase when additional data contradict existing data. Sampling wastewater data from the entire population every day is expected to further reduce daily uncertainty by 14.6% (or 29.4% when compared to estimates from reported case data only), representing a plausible bound on the expected reduction in $R_{t}$ uncertainty achievable through wastewater sampling.

Missing wastewater data may (once collected) differ systematically from observed data, for example if existing sampling is biased towards sites expected to yield more reliable measurements. In this case, we might wish to allow the variance of the observation distribution for the wastewater data to be larger in the hypothetical-data setting than in the observed data. To maintain coherence and ensure more data reduces our model uncertainty, we would also need to include this assumption in the model itself, or we risk using a predictive model that is more diffuse than implied by our model (as highlighted in Principle 4).

## Related frameworks

Our decision-theoretic approach unifies multiple popular methodologies. We describe the conceptual connections between these methods and include pointers to literature with the technical details.

The **VOI** framework, widely used in health economics, measures the expected reduction in loss from obtaining additional data.^17^^-^^19^^,^^41^^-^^43^ The central concept of the *expected value of sample information* (EVSI) is equivalent to our expected uncertainty reduction (Equation 6) in a standard Bayesian setting. Our framework extends the VOI literature beyond the traditional Bayesian setting by allowing for a wider class of predictive distributions and models. The VOI framework is also applied to decision problems, where the loss function represents the cost of a decision based on the model. Our framework adds the interpretation that the minimised expected cost is a direct measure of uncertainty about that decision.

**Bayesian experimental design** and **active learning** focus on data collection strategies, arguing that experiments should be designed to maximise their expected utility, commonly framed as the *expected information gain* (EIG).^20^^,^^44^ The EIG about a quantity *z* from a proposed experiment is the expected reduction in posterior entropy, equivalent to our framework under a log-loss function. By Principle 4, an experiment is only guaranteed to reduce uncertainty if we model the data consistently. Case et al.^6^ apply these principles in epidemiology for tick surveillance design, and our framework encourages viewing their bespoke risk-based loss function as the relevant measure of uncertainty in that setting.

**Fisher information** approaches can also be captured. For example, Parag et al.^15^ used Fisher information to quantify the information in noisy epidemic curves, which is approximately equivalent to the inverse-variance of the maximum likelihood estimate. Under our framework, this corresponds to using a quadratic loss function and measuring uncertainty via the variance of a point estimate.

Finally, **scoring rules** have gained popularity in epidemiology for measuring the quality of probabilistic estimates.^7^^,^^23^^,^^29^^,^^45^^,^^46^ Estimation of future data is distinct from estimation of model parameters in that we eventually observe the data, and thus can calculate the loss (score) associated with a given prediction. In our framework, applying a scoring rule to each forecast-outcome pair is an operationalization of the loss, giving a setting in which uncertainty can be empirically estimated.

## Discussion

We have presented a decision-theoretic framework for conceptualising and quantifying uncertainty in epidemiological models. Starting from the idea that uncertainty arises from missing data,^13^ our approach directly links theory to practical application and yields a separation of reducible and irreducible uncertainty. We then demonstrated that coherence ensures collecting additional data will reduce uncertainty on average, reinforcing missing data as the source of uncertainty. The framework was illustrated using a published model of SARS-CoV-2 wastewater surveillance in Aotearoa New Zealand,^39^ where the practical limit of wastewater data in reducing $R_{t}$ uncertainty was estimated.

By connecting, extending, and generalising existing frameworks from adjacent fields, including VOI analysis, Bayesian experimental design, and scoring rules, we aim to make these powerful ideas more accessible to epidemiologists. Our framework broadens the applicability of these tools, clarifying how divergent perspectives on uncertainty often overlap. While our focus has been on epidemiology, the framework is applicable to a wider range of fields, including weather forecasting, economics, and ecology,^7^^,^^47^ with ideas from these fields also informing aspects of our framework.

This work lays the foundation for potentially high-impact future research, including (i) application-specific loss functions, (ii) general coherent predictive models, and (iii) the evaluation of model specification. For (i), adopting losses that reflect the decision context could lead to more relevant notions of uncertainty, particularly in settings where being above a certain threshold (such as $R_{t}> 1$) is of greater concern than being below it.^48^ For (ii), while we focused on Bayesian examples, the framework supports a broader class of models, including black-box machine-learning models. Active research in the foundations of predictive inference is expanding the class of models for which coherence can be formally verified, an important step towards applying our framework to non-Bayesian methods^.13^^,^^28^^,^^37^^,^^49^ We note that determining whether a given model satisfies coherence in practice is often model-specific and can require tailored diagnostic methods. For (iii), as epidemiological models are typically fit to a single data realisation, model structure plays a greater role in producing estimates than in many other fields. There is consequently greater potential for model misspecification, addressable by extending our framework using reference distributions,^9^ parametric extensions,^50^ and model averaging.^50^^,^^51^

In many epidemiological applications, the predictive distribution for $y_{miss}$ will take the form of a simulator. The expected uncertainty reduction can then be estimated by sampling from this simulator, re-fitting the model, calculating the uncertainty, and repeating multiple times. This can be computationally expensive, and existing techniques such as Gaussian process surrogates or neural density estimators^12^^,^^52^^,^^53^ could accelerate this.

A practical advantage of the loss-based approach is that it grounds uncertainty in problem-specific, interpretable terms. Under quadratic loss, for instance, uncertainty is the expected squared error of the best estimate, a quantity that can be conveyed as “how far off our best guess is likely to be.” Under alternative, tailored losses, uncertainty can be expressed in units familiar to decision-makers, such as the expected monetary cost of acting on imperfect information. For example, planning Intensive Care Unit (ICU) capacity for an anticipated epidemic wave involves a clear cost asymmetry: each unmet bed costs more than each unused bed (in both monetary and human terms). Choosing a loss function that reflects this asymmetry expresses uncertainty about peak demand as the expected cost of acting on the current best estimate. The expected gain from additional surveillance is the reduction in this cost, which can be weighed against the cost of collecting it. This is the central question of value-of-information analysis in healthcare,^18^^,^^19^ applied in this exact setting for early COVID-19 ICU preparedness by Eze et al.^54^ By linking the mathematical measure of uncertainty to its real-world meaning, we can better communicate uncertainty to policymakers and the public, facilitating informed decision-making.

By framing uncertainty as a consequence of estimation under incomplete information, we provide a theoretically grounded and practical approach to uncertainty quantification in epidemiological modelling, particularly useful for planning studies and directing surveillance efforts. Our framework supports consistent, reproducible, and policy-relevant model-based inference in infectious disease epidemiology.

## Supplementary Material

Web_Material_kwag154

## Data Availability

All code and data to reproduce the wastewater application are available at: https://github.com/nicsteyn2/DecisionTheoreticEpi. A supplementary file (consisting of Appendices S1, S2, and S3) containing formal proofs, derivations, and full technical details is available alongside the main text.
