# Supplementary material for: A decision-theoretic framework for uncertainty quantification in epidemiological modelling
Source: Am J Epidemiol. 2026 Jul 8;195(11):2783–92. doi: 10.1093/aje/kwag154 (PMC13733546; doi:10.1093/aje/kwag154)
Supplement: Supplementary file 1 [file web_material_kwag154.zip › Web_Material_kwag154.pdf]

Supplementary material for: A decision-theoretic framework for uncertainty quantification in epidemiological modelling

Nicholas Steyn[1], Cathal Mills[1,2], Vik Shirvaikar[1], Freddie Bickford Smith[1], Christl A Donnelly[1,2], and Kris V Parag[3,4]

[1]Department of Statistics, University of Oxford
[2]Pandemic Science Institute, University of Oxford
[3]Department of Engineering, King's College London
[4]MRC Centre for Global Infectious Disease Analysis, Imperial College London

Included materials

S1 Derivation of common uncertainty measures **2**

S2 Coherence: formal definition and proof **3**

S3 Technical details of the wastewater surveillance application **4**

S1 Derivation of common uncertainty measures

The main text defines uncertainty as the minimised expected loss:

$$h\left[p(z|y_{obs})\right] = \min_{a \in \mathcal{A}} E_{p(z|y_{obs})}[\ell(a,z)] = E_{p(z|y_{obs})}[\ell(a^*,z)], \tag{1}$$

where a^* is the Bayes-optimal estimate. Here we show that two common loss functions yield two familiar uncertainty measures.

Quadratic loss yields posterior variance

Suppose we make a point estimate $a \in \mathbb{R}$ of $z \in \mathbb{R}$ under the quadratic (squared-error) loss $\ell(a,z) = (a-z)^2$. The expected loss is:

$$E_{p(z|y_{obs})}[(a-z)^2]. \tag{2}$$

To find a^*, we differentiate with respect to a and set the result to zero:

$$\frac{d}{da} E_{p(z|y_{obs})}[(a-z)^2] = 2\, E_{p(z|y_{obs})}[a-z] = 2(a - E_{p(z|y_{obs})}[z]) = 0, \tag{3}$$

giving $a^* = E_{p(z|y_{obs})}[z]$, the posterior mean. Substituting back:

$$\begin{aligned} h\left[p(z|y_{obs})\right] &= E_{p(z|y_{obs})}\left[\left(E_{p(z|y_{obs})}[z] - z\right)^2\right] \\ &= \mathrm{Var}_{p(z|y_{obs})}(z). \end{aligned} \tag{4}$$

That is, under quadratic loss, uncertainty is measured by the posterior variance.

Log-loss yields posterior entropy

Suppose we make a probabilistic estimate: we choose a probability distribution q over z (so the action space is the set of probability distributions on $\mathcal{Z}$), under the log-loss $\ell(q,z) = -\log q(z)$. The expected loss is:

$$E_{p(z|y_{obs})}[-\log q(z)]. \tag{5}$$

This is the cross-entropy between $p(z|y_{obs})$ and q. By Gibbs' inequality, the cross-entropy is minimised when $q = p(z|y_{obs})$, so $a^* = p(z|y_{obs})$: the optimal probabilistic estimate is the posterior distribution itself. Substituting back:

$$\begin{aligned} h\left[p(z|y_{obs})\right] &= E_{p(z|y_{obs})}\left[-\log p(z|y_{obs})\right] \\ &= \mathrm{H}\left[p(z|y_{obs})\right], \end{aligned} \tag{6}$$

the entropy of the posterior distribution. That is, under log-loss, uncertainty is measured by the posterior entropy.

S2 Coherence: formal definition and proof

This appendix provides the formal definition of coherence and a proof that coherence implies a non-negative expected uncertainty reduction, as stated in Principle 4 of the main text. We also discuss its connection to Bayesian inference.

Definition

$p(y_{miss}|y_{obs})$ is *coherent* with the fitted model $p(z|y_{obs})$ if both are marginal distributions of the same joint distribution $p(z, y_{all})$, implying:

$$p(z|y_{obs}) = \int_{\mathcal{Y}} p(z|y_{all})\, p(y_{miss}|y_{obs})\, dy_{miss}, \tag{7}$$

where $\mathcal{Y}$ is the space of possible values of y_{miss}. This condition says that the predictive distribution and the fitted model make the same assumptions about the missing data. Equivalently, the posterior distribution for z given only y_{obs} can be recovered by averaging the updated posterior distribution $p(z|y_{all})$ over all possible values of the missing data under $p(y_{miss}|y_{obs})$. Consequently, the expected value of z should not change after including missing data sampled from $p(y_{miss}|y_{obs})$.

Proof that coherence implies non-negative expected uncertainty reduction

Define a^* as the Bayes-optimal estimate of z prior to observing y_{miss} (but after observing y_{obs}, which we condition on throughout). After observing y_{miss}, the Bayes-optimal estimate may change. Since a^* need not be optimal under the updated distribution $p(z|y_{all})$, we have the following inequality for any realisation of y_{miss}:

$$\min_a \mathrm{E}_{p(z|y_{all})}\left[\ell(a, z)\right] \leq \mathrm{E}_{p(z|y_{all})}\left[\ell(a^*, z)\right]. \tag{8}$$

Taking expectations of both sides over $p(y_{miss}|y_{obs})$:

$$\mathrm{E}_{p(y_{miss}|y_{obs})}\left\{\min_a \mathrm{E}_{p(z|y_{all})}\left[\ell(a, z)\right]\right\} \leq \mathrm{E}_{p(y_{miss}|y_{obs})}\left\{\mathrm{E}_{p(z|y_{all})}\left[\ell(a^*, z)\right]\right\}. \tag{9}$$

The left-hand side is the expected uncertainty after collecting y_{miss}, i.e. $\mathrm{E}_{p(y_{miss}|y_{obs})}[h(p(z|y_{all}))]$.

For the right-hand side, we apply the law of total expectation. By coherence (Equation 7), averaging $p(z|y_{all})$ over $p(y_{miss}|y_{obs})$ recovers $p(z|y_{obs})$. Therefore:

$$\mathrm{E}_{p(y_{miss}|y_{obs})}\left\{\mathrm{E}_{p(z|y_{all})}\left[\ell(a^*, z)\right]\right\} = \mathrm{E}_{p(z|y_{obs})}\left[\ell(a^*, z)\right] = h\left[p(z|y_{obs})\right]. \tag{10}$$

Combining gives:

$$\mathrm{E}_{p(y_{miss}|y_{obs})}\left[h\left(p(z|y_{all})\right)\right] \leq h\left(p(z|y_{obs})\right), \tag{11}$$

so the expected uncertainty reduction is non-negative.

Equality holds if and only if a^* remains optimal for every realisation of y_{miss}, which occurs if and only if z is conditionally independent of y_{miss} given y_{obs}. That is, the expected uncertainty reduction is zero only when the

missing data provide no new information about z.

S3 Technical details of the wastewater surveillance application

This appendix provides additional details of the uncertainty quantification procedure applied to the SARS-CoV-2 wastewater surveillance model for Aotearoa New Zealand, as described in Section 3 of the main text. The underlying epidemiological model is the state-space model of Watson et al. [1], which jointly estimates the instantaneous reproduction number R_t, case ascertainment rate, and infection incidence from reported case data and wastewater concentration data using sequential Monte Carlo methods. We refer the reader to that paper for full model details. Here, we describe how our framework is applied to that model.

We assume decision-makers use point estimates of R_t with a quadratic loss function deemed suitable, and therefore use the posterior variance of R_t as our measure of uncertainty.

Uncertainty reduction from observed wastewater data

To assess the benefit of incorporating already-observed wastewater data, we compute the daily uncertainty reduction (Equation 5 in the main text). In this setting, y_{miss} are the daily wastewater observations $W_{1:T}$, and y_{obs} are the daily reported case data $C_{1:T}$. The uncertainty reduction in R_t at time-step $t \in 1, 2, \ldots, T$ is given by $\mathrm{Var}_{p(R_t|C_{1:T})}(R_t) - \mathrm{Var}_{p(R_t|C_{1:T},W_{1:T})}(R_t)$, where $p(R_t|C_{1:T})$ is the posterior distribution of R_t given only the reported case data, and $p(R_t|C_{1:T}, W_{1:T})$ is the posterior distribution of R_t given both the reported case data and the wastewater data. Each posterior distribution is obtained by fitting the model to the respective datasets using the inference procedure of Watson et al. [1].

Expected uncertainty reduction from expanding wastewater surveillance

To estimate the expected uncertainty reduction in R_t from daily wastewater sampling of the entire population, we first fit the model to the observed data $C_{1:T}$ and $W_{1:T}$, obtaining the joint posterior distribution over infection incidence $I_{1:T}$ and the vector of all other model parameters $\boldsymbol{\theta}$ (including observation-model and epidemiological parameters), denoted $p(I_{1:T}, \boldsymbol{\theta} \mid C_{1:T}, W_{1:T})$. We then simulate hypothetical missing wastewater data $W'_{1:T}$ by assuming the daily catchment population for the new data equals the population not already covered by existing wastewater sampling, and sampling from the posterior predictive distribution:

$$p(W'_{1:T}|C_{1:T}, W_{1:T}) = \iint p(W'_{1:T}|I_{1:T}, \boldsymbol{\theta})\, p(I_{1:T}, \boldsymbol{\theta}|C_{1:T}, W_{1:T})\, dI_{1:T}\, d\boldsymbol{\theta}. \tag{12}$$

In practice, this is done by drawing samples $(I_{1:T}^{(s)}, \boldsymbol{\theta}^{(s)})$ from the joint posterior and, for each sample, simulating wastewater data $W_{1:T}^{\prime(s)}$ from the observation model $p(W'_{1:T}|I_{1:T}^{(s)}, \boldsymbol{\theta}^{(s)})$.

For each simulated dataset $W_{1:T}^{\prime(s)}$, we construct the "expanded" wastewater dataset by taking the daily population-weighted average of the observed wastewater data $W_{1:T}$ and the simulated data $W_{1:T}^{\prime(s)}$, representing what we would observe if sampling covered the entire national population. We then re-fit the model to the reported cases and this combined wastewater dataset, and compute the resulting posterior variance of R_t. Repeating this $S = 100$ times

and averaging yields a Monte Carlo estimate of the expected uncertainty after expanding surveillance:

$$\mathrm{E}_{p(W'_{1:T}|C_{1:T},W_{1:T})}\left[\mathrm{Var}_{p(R_t|C_{1:T},W_{1:T},W'_{1:T})}(R_t)\right] \approx \frac{1}{S}\sum_{s=1}^{S}\mathrm{Var}_{p(R_t|C_{1:T},W_{1:T},W'^{(s)}_{1:T})}(R_t). \tag{13}$$

The expected uncertainty reduction (Equation 6 in the main text) is then the difference between the posterior variance under the current data and this Monte Carlo average.

Coherence

This procedure uses the posterior predictive distribution implied by the fitted model as the predictive distribution for the missing data. As the model is Bayesian, this posterior predictive distribution is automatically coherent with the fitted model (Appendix S2), guaranteeing that the expected uncertainty reduction is non-negative. As shown in Figure 6-D of the main text, individual realisations of $W'_{1:T}$ may increase uncertainty on certain days, but the average across realisations will not.

We note that missing wastewater data may, once collected, differ systematically from observed data—for example, if existing sampling is biased towards sites expected to yield more reliable measurements. In this case, one might wish to allow the variance of the observation distribution for the hypothetical wastewater data to be larger than for the observed data. To maintain coherence and ensure that including additional data reduces model uncertainty on average, this assumption would also need to be incorporated into the model itself.

References

[1] L. M. Watson et al. "Jointly Estimating Epidemiological Dynamics of Covid-19 from Case and Wastewater Data in Aotearoa New Zealand". In: *Communications Medicine* 4.1 (2024), pp. 1–9. ISSN: 2730-664X. DOI: 10.1038/s43856-024-00570-3.
